# Muscle hypertrophy in hypoxia with inflammation is controlled by bromodomain and extra-terminal domain proteins

**DOI:** 10.1038/s41598-017-12112-0

**Published:** 2017-09-21

**Authors:** Clovis Chabert, Saadi Khochbin, Sophie Rousseaux, Rebecca Furze, Nicholas Smithers, Rab Prinjha, Uwe Schlattner, Christophe Pison, Hervé Dubouchaud

**Affiliations:** 1 Univ. Grenoble Alpes, Inserm, Laboratoire de Bioénergétique Fondamentale et Appliquée (LBFA), Grenoble, 38000 France; 2 Univ. Grenoble Alpes, Inserm, CNRS, Institute for Advanced Biosciences (IAB), Grenoble, 38000 France; 3 Epigenetics DPU, Immuno-Inflammation Therapy Area, Medicines Research Centre, GlaxoSmithKline R&D, Stevenage, SG1 2NY, England UK; 4 Univ. Grenoble Alpes, Inserm, CHU des Alpes, Laboratoire de Bioénergétique Fondamentale et Appliquée (LBFA), Grenoble, 38000 France

## Abstract

Some of the Chronic Obstructive Pulmonary Disease (COPD) patients engaged in exercise-based muscle rehabilitation programs are unresponsive. To unravel the respective role of chronic hypoxia and pulmonary inflammation on soleus muscle hypertrophic capacities, we challenged male Wistar rats to repeated lipopolysaccharide instillations, associated or not with a chronic hypoxia exposure. Muscle hypertrophy was initiated by bilateral ablation of soleus agonists 1 week before sacrifice. To understand the role played by the histone acetylation, we also treated our animals with an inhibitor of bromodomains and extra terminal proteins (I-BET) during the week after surgery. Pulmonary inflammation totally inhibited this hypertrophy response under both normoxic and hypoxic conditions (26% lower than control surgery, p < 0.05), consistent with the S6K1 and myogenin measurements. Changes in histone acetylation and class IIa histone deacetylases expression, following pulmonary inflammation, suggested a putative role for histone acetylation signaling in the altered hypertrophy response. The I-BET drug restored the hypertrophy response suggesting that the non-response of muscle to a hypertrophic stimulus could be modulated by epigenetic mechanisms, including histone-acetylation dependant pathways. Drugs targeting such epigenetic mechanisms may open therapeutic perspectives for COPD patients with systemic inflammation who are unresponsive to rehabilitation.

## Introduction

Chronic obstructive pulmonary disease (COPD) is characterized by a progressive and persistent airflow limitation^1^. This condition is usually associated with an enhanced chronic inflammatory response to noxious particles or gases in the airways and the lungs. Body composition abnormalities, especially muscle wasting, are of paramount importance among comorbidities due to their major role in vital prognosis^2^. Persistent systemic inflammation as a spill-of lung response to environmental stresses has been proposed to explain, in part, the observed muscle alterations^3^, survival rate^4^ and comorbidities^5^. In particular, there is a cross-talk between chronic pulmonary inflammation (PI) and chronic hypoxia (CH)^6,7^ which could contribute to the muscle alterations observed in COPD^8^. For example, synthesis of oxidative myosin heavy chain is inhibited by inflammation^9^. Additionally, proteosynthesis signaling pathways in plantaris muscle of rats exposed to CH transiently decreased after an overload surgery^10^.

To reverse muscle wasting in COPD, pulmonary rehabilitation based on a multimodal approach including nutritional intervention and exercise training has been suggested^2,11–14^. Unfortunately, up to 30% of patients do not respond to such programs^15,16^ while others show lower responses compared to healthy subjects^17^, even after lung transplantation^18^. To explain this muscle unresponsiveness, epigenetic alterations have been proposed, such as changes in the level of the histone acetylation pattern, which could impact the gene response^19^. Protein modifications linked to the acetylation level have been shown in the lung and in the muscle of COPD patients^17,20,21^. Moreover, CH and PI are well known to induce epigenetic alterations through changes in transcription and/or enzymatic activity of histone deacetylases (HDAC)^22–24^, which could be involved in muscle atrophy^25^ or in alterations of the proteosynthesis pathways^26^. Reversible acetylation of histones, catalysed by histone acetyltransferases (HATs) and histone-deacetylases (HDACs), has been characterized as an important signal controlling the state of gene expression^27^, and also constitutes a now well-established target for a variety of therapeutic molecules.

Bromodomains (Brd) which are very conserved and specific structural motifs present in one or more copies in 46 distinct human proteins (61 different Brds have been identified so far), are essential relays for transmitting and/or interpreting signals initiated by histone acetylation that recognise acetyl-labels on histones and non-histone proteins^28^. Brd containing proteins have a variety of critical chromatin regulatory functions such as HAT, ATPase, and histone lysine methyltransferase activity^29^. Indeed, inhibition of interactions between Brd-proteins and acetylated histones could therefore constitute an elegant strategy to “cut at the source”, the histone acetylation-signaling systems. Several cell permeable small molecules have been recently shown to efficiently interfere with the binding of a family of specific regulators of chromatin and transcription, known as Brd and extra terminal (BET) family^30^. Recent investigations showed the role of BRD4, a member of the BET family, in the regulation of inflammatory genes and prompted investigators to use BET inhibitors as a novel anti-inflammatory approach^24^. Additionally, BET proteins have been also involved in mechanisms controlling skeletal muscle wasting and mass determination^31^.

Our hypothesis is that a lack of appropriate response of muscles to exercise rehabilitation in the context of PI and CH, such as in COPD patients, may involve alterations in histone acetylation patterns. Therefore our aim was to unravel the respective roles of PI and CH in the soleus muscle hypertrophic capacities induced by a bilateral ablation of its agonist muscles (plantaris and gastrocnemius). This overload model allowed the investigation of several major signaling pathways and epigenetic actors involved in proteosynthesis/lysis, with a specific focus on the role of BET proteins.

## Material and Methods

### Animals

**Figure 1:**
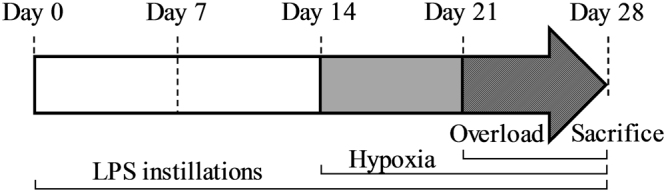
Chronogram of experimentation for pulmonary inflammation induced by lipopolysaccharides (LPS), chronic hypoxia and muscle overload surgery.

Adult Wistar male rats (4 month-old, 442 ± 28 g) were used in this study. All animal studies were ethically reviewed and carried out in accordance with European Directives 86/609/EEC, 2010/63/UE and the GSK policy on the care, welfare and treatment of animals. All the procedures were approved by the ethics committee (Cometh Grenoble) affiliated to the animal facility of the university (D3842110001), and agreed by the French Ministry of Research (168_LBFA-U1055 and 345_LBFA-U1055). A summary of the experimental design is reported in Fig. 1.

### Pulmonary inflammation

A chronic pulmonary inflammation was induced by bi-weekly lipopolysaccharides (LPS) intracheal administration to the animals at 1 mL/kg of body weight (E.coli, serotype O55:B5; Sigma-Aldrich Chemical Co.™, S^t^ Louis, Missouri, USA in NaCl 0.9% at 0.4 mg/mL) for 4 weeks as previously described^32^.

### Chronic Hypoxia

During the last 14 days of the protocol, animals from hypoxia groups were exposed to a chronic normobaric hypoxia (FiO_2_: 10%). The hypoxia chamber was opened 45-60 minutes *per* day to weight the animals, the food, and to perform LPS administration.

### Muscle overload model

Muscle hypertrophy was induced by a bilateral ablation of the agonist muscles of the soleus as described by Morioka *et al*.^33^ and treated with Paracetamol to avoid pain. Sham operations were also performed under the same conditions as described in supplementary materials and methods.

### Drug administration

Bromodomain and extra-C-terminal domain inhibitor 151 (I-BET151), also referenced as GSK1210151 (GlaxoSmithKline™, London, England), was administered by daily oral gavage (10 mg/mL/kg in methyl cellulose 400cp 1% in H_2_O) during the week post-surgery. The untreated animals received the same volume of vehicle.

### Experimental design of animal study

To characterize the response of the soleus muscle to the hypertrophic stimulus, animals were assigned to one of the five following groups: normoxia control (NC), normoxia + surgery (NS), normoxia + surgery + inflammation (NSI), chronic hypoxia + surgery (HS) and chronic hypoxia + surgery + inflammation (HSI).

To study the effects of the BET inhibitor, animals were also subjected to our model and treated with vehicle or iBET from day 21 to 28. Animals were assigned to one of the six following groups: normoxia control + vehicle (NCv), normoxia control + I-BET151 (NCiB), normoxia + surgery + vehicle (NSv), normoxia + surgery + I-BET151 (NSiB), chronic hypoxia + surgery + inflammation + vehicle (HSIv), and chronic hypoxia + surgery + inflammation + I-BET151 (HSIiB).

### Tissue sampling

Animals were euthanized with a dose of pentobarbital at 50 mg/kg of body weight. Then, the soleus muscles were dissected, weighed and directly frozen in liquid nitrogen. Hypertrophic capacity was determined by the ratio between soleus weight normalized with tibia length and the mean value obtained in the control group and expressed in hypertrophy percentage.

### Protein isolation and immunoblottings

Contents for the ribosomal protein S6 kinase beta-1 (S6k1), phospho-S6k1^thr389^, the extracellular signal-regulated kinases 1-2 (Erk 1-2), phospho-Erk 1-2^thr202/tyr204^, the protein kinase B also known as Akt, phospho-Akt^ser473^, and Myogenin were measured by Western blot as described in details in the supplemental data. Due to the variety of experimental treatments (hypoxia, surgery and inflammation), we were unable to find a stable protein as a loading control. To circumvent this problem, membranes were then colored using Ponceau S staining to ensure that protein amounts were similar in each well (Supplementary Figure 1). Blot signals were then normalised with Ponceau S staining of the whole lane.

### Histones extraction and acetylation measurement

Histones were extracted by trichloroacetic acid precipitation as in Shechter *et al*.^34^. Sodium butyrate 10 mM was added to maintain the acetylation of lysines in the samples. Acetylated and total levels of histone H3 were determined by Western blot, using a pan acetyl antibody which shows a preference for acetylated histone H33 (#9441 S, Cell signaling Technology, RRID:AB_331805), normalized by the total amount of histone 3^35^ (#9715, Cell Signaling Technology, RRID:AB_331563). Antibodies were incubated overnight at 4 °C after a 1h-membrane saturation in TBS, pH 7.4, 0.01% (v/v) tween 20, 5% (w/v) BSA. Anti-rabbit secondary antibodies coupled with HRP diluted at 1:3000 (anti-rabbit NA934 (RRID:AB_772206), GE Healthcare™, Little Chalfont, England) was then incubated for 1 hour at room temperature. Reactions were revealed as described in the supplemental data. Membranes were stripped between primary antibody incubation. A pooled sample was systematically loaded on each gel to allow the comparison between gels.

### RNA extraction and RT-qPCR

Total RNA was purified in TRIzol (ThermoFisher Scientific™, Waltham, Massachusetts, USA). Reverse transcription of mRNA was performed with Superscript III kit (Invitrogen, Carlsbad, California, USA). Relative contents of target mRNA were determined by real-time PCR using a LightCycler (Roche Applied Science™, Bâle, Switzerland) with the primers reported in supplementary materials and methods (Supplementary Table 1). Quantification of PCR products was done using the (1 + E)^−ΔΔCT^ method^36^. Hypoxanthine phosphoribosyltransferase-1 (Hprt1), was used as a housekeeping gene.

### Statistical analysis

All the data are presented as mean ± SEM. When data were normally distributed, a two-way ANOVA was performed to determine the effects of environment (normoxia *vs*. chronic hypoxia) and inflammation (without administration *vs*. LPS administration). If significant, the omnibus test of variance was followed by a Holm-Sidak *post-hoc*. For non-parametric data, a Tukey test was used to determine the nature of the effect. Impact of the I-BET151 where tested with a one-way ANOVA followed by a Holm-Sidak *post-hoc* if significant. Correlation coefficients were calculated with a Pearson product moment.

## Results

### Soleus muscle hypertrophy in response to surgical overload

**Figure 2:**
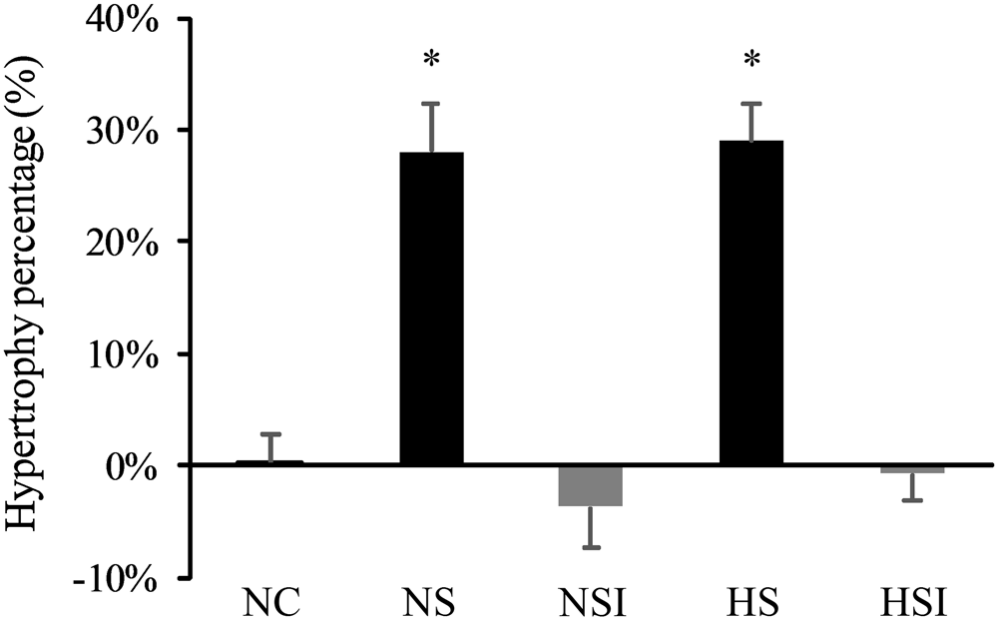
Effects of pulmonary inflammation and hypoxia exposure on the hypertrophic capacity of soleus muscle subjected to an overload surgery. Hypertrophic capacity was determined by the ratio between soleus weight normalized with tibia length and the mean of the control group. (n = 7–8; Mean ± SEM); NC: Normoxia Control; NS: Normoxia + Surgery; NSI: Normoxia + Surgery + Inflammation; HS: Hypoxia + Surgery; HSI: Hypoxia + Surgery + Inflammation. *Different *vs*. NC, NSI and HSI, p < 0.05.

Soleus muscle hypertrophic capacity induced by functional overload generated by surgical removal of agonist muscles was studied in response to the effect of CH and/or PI (Fig. 1). Surgery induced muscle hypertrophy as measured by a significant increase in soleus weight in both normoxia (NS) and hypoxia (HS) as compared to the control group (NC; + 28% and + 29% respectively, p < 0.05, Fig. 2). However, in animals subjected to PI, the hypertrophic capacity of soleus was totally inhibited in both normoxia (−3.64%) and hypoxia (−0.72%) when compared to the control group (Fig. 2). Hypoxia exposure was associated with lower final body weight compared to normoxia groups in both sets of experiments (supplementary Table 2A and B). However tibia length was not significantly different between all the groups.

This first experiment therefore suggests that PI dramatically inhibits the soleus hypertrophic capacity, regardless the oxygen availability.

### Proteosynthesis/proteolysis balance

**Figure 3:**
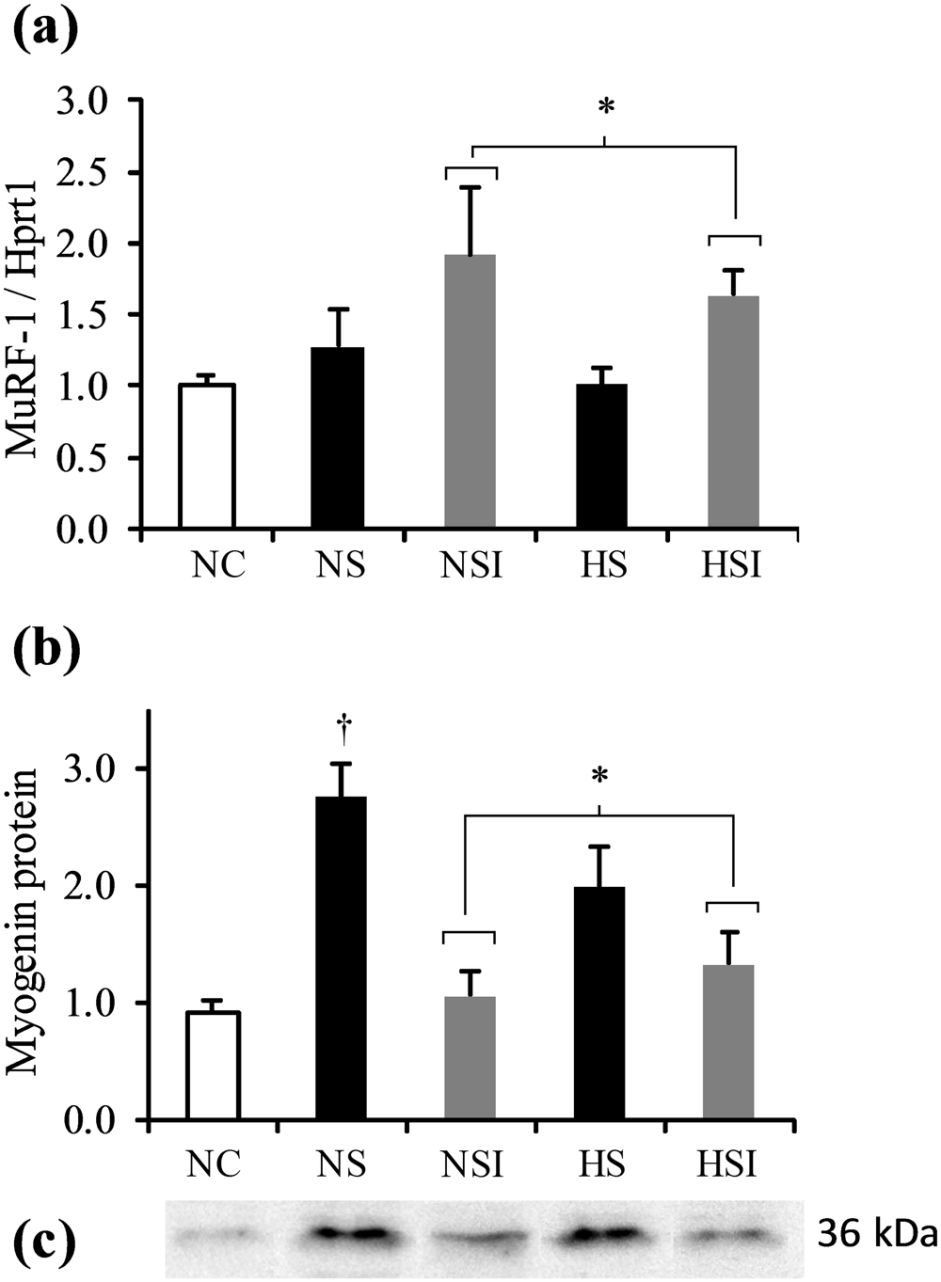
Effects of pulmonary inflammation and hypoxia exposure on the MuRF-1 mRNA contents determined by RT-PCR (**a**) and the myogenin contents (**b**) determined by Western blot (**c**), in soleus muscle subjected to an overload surgery. (n = 5–6; Mean ± SEM); Hprt1 was used as a housekeeping gene. NC: Normoxia Control; NS: Normoxia + Surgery; NSI: Normoxia + Surgery + Inflammation; HS: Hypoxia + Surgery; HSI: Hypoxia + Surgery + Inflammation. †NS *vs*. NC, p < 0.05; *global effect of inflammation (NS + HS) *vs*. (NSI + HSI), p < 0.05. Blot image has been cropped to the area of interest. The full-length blot is presented in Supplementary Figure 3.

To explain the observed alterations in muscle mass, we then investigated the proteosynthesis and proteolysis balance through selected indices. The levels of MuRF-1 mRNA and of myogenin protein in soleus muscle were used as indicators of the proteolysis and proteosynthesis pathways, respectively (Fig. 3a and b). When comparing the different treatments of the overload model, MuRF-1 mRNA content was significantly higher only in animals with additional PI (+47%, NSI + HSI *vs*. NS + HS, p < 0.05; Fig. 3a). Surgery for the overload model (NS *vs*. NC) increased myogenin (Fig. 3b) but not MuRF-1 mRNA levels (Fig. 3a), consistent with increased proteosynthesis in hypertrophy. The opposite was found for myogenin protein content, which was significantly higher only in animals without PI (+100%, NS + HS *vs*. NSI + HSI, p < 0.05), while it remained at control levels in groups with PI (Fig. 3b). No significant differences were observed for MuRF-1 mRNA or myogenin content in the overload model when comparing normoxia with hypoxia, in both absence and presence of PI.

**Figure 4:**
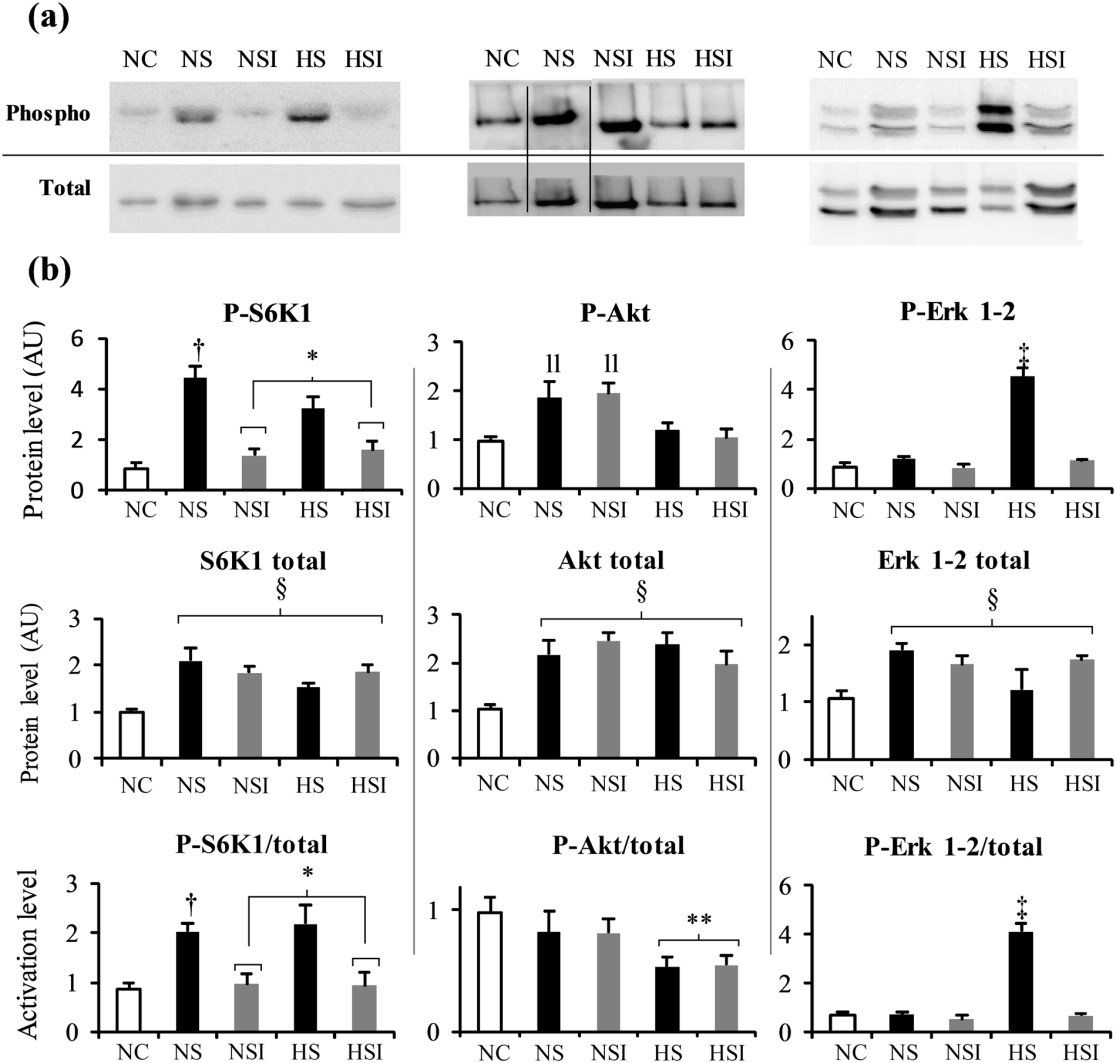
Effects of pulmonary inflammation and hypoxia exposure on markers of the proteosynthesis pathways in soleus muscle subjected to an overload surgery. Phosphorylated (P-) and total form of S6K1 (70 kDa), Akt (60 kDa) and Erk 1-2 (upper band: Erk 1 (44 kDa, lower band: Erk 2 (42 kDa)) were determined by Western blot (a and b). Activation level were calculated as the Phosphorylated/Total ratio. (n = 5–7; Mean ± SEM); NC: Normoxia Control; NS: Normoxia + Surgery; NSI: Normoxia + Surgery + Inflammation; HS: Hypoxia + Surgery; HSI: Hypoxia + Surgery + Inflammation. ^†^NS *vs*. NC, p < 0.05; *global effect of inflammation (NSI + HSI) *vs*. (NS + HS), p < 0.05; ^‡^different *vs*. all others, p < 0.05; ll: different vs. NC, HS and HSI, p < 0.05; **global effect of hypoxia (HS + HSI) *vs*. (NS + NSI), p < 0.05; ^§^(NS + NSI + HS + HSI) *vs*. NC, p < 0.05. Blot images have been cropped to the area of interest. The full-length blot are presented in Supplementary Figure 3.

We further studied pathways involved in proteosynthesis, through phosphorylation of ribosomal protein S6 kinase 1 (S6K1) as a suitable readout of the pro-hypertrophic mTOR pathway (Fig. 4a,b, left panels), and phosphorylation of Akt and Erk, since activation of these two pathways can act as upstream trigger of mTOR. We focussed on these proteins as they correspond to the beginning and the end of the corresponding signaling pathway. Expression of all these three proteins was increased by the surgery overload (NS + NSI + HS + HSI *vs*. NC, p < 0.05). The S6K1 phosphorylation state (P-S6K1/total S6K1 ratio) increased by surgery (+104%, NS *vs*. NC, +119%, HS *vs*. NC, p < 0.05), but was significantly higher only in absence of PI under normoxia and hypoxia (NS *vs*. NSI, HS *vs*. HSI, p < 0.05), while it remained at control levels in PI groups. This response pattern correlates very well with those seen for muscle hypertrophy and myogenin levels shown above (Figs 2 and 3b). The Akt phosphorylation state (P-Akt/total Akt) did not change upon surgery in normoxia, only hypoxia led to a significant decrease, while PI had no effect. However, when total Akt phosphorylation is considered, the surgery induced an increase under normoxia (+91% NS *vs*. NC and +100% NSI *vs*. NC, p < 0.05), not present under hypoxia. The pattern of the Erk 1-2 phosphorylation state partially differs from Akt; while it was also unchanged upon surgery and normoxia, it was very much higher in hypoxia, but only in absence of PI (+421% HS *vs*. NC, p < 0.05).

From these experiments it appears that proteolysis is not unbalanced in the same way by CH and PI. Measurement of S6K1 suggests a strong inhibition of this pathway by PI, whereas CH effects could be compensated by the Erk 1-2 pathway.

### Histone acetylation and class IIa HDACs expression

**Figure 5:**
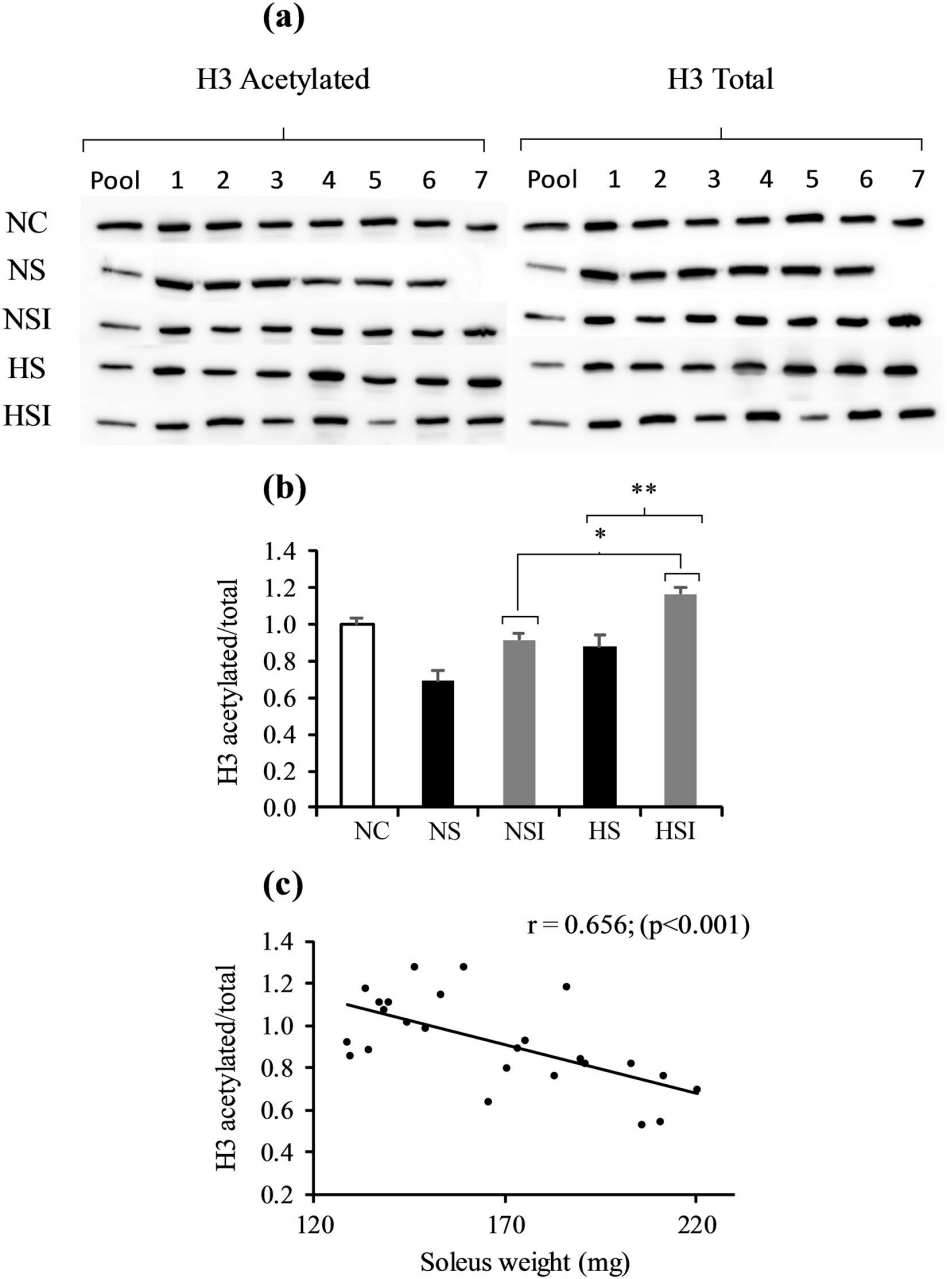
Effects of pulmonary inflammation and hypoxia exposure on histone H3 acetylation levels in soleus muscle subjected to an overload surgery. Panel a shows blots of the acetylated and the total form of histone 3. Numbers represent different animals. Western blots with antibodies against acetyl-lysine recognizing preferentially acetylated histone H3 and total histone H3 were quantified and normalized (panel b) and related to the soleus muscle weight (panel c). (n = 6-7; Mean ± SEM); NC: Normoxia Control; NS: Normoxia + Surgery; NSI: Normoxia + Surgery + Inflammation; HS: Hypoxia + Surgery; HSI: Hypoxia + Surgery + Inflammation. *Global effect of inflammation (NSI + HSI) *vs*. (NS + HS), p < 0.05; **global effect of hypoxia (HS + HSI) *vs*. (NS + NSI), p < 0.05. Blot images have been cropped to the area of interest.

**Figure 6:**
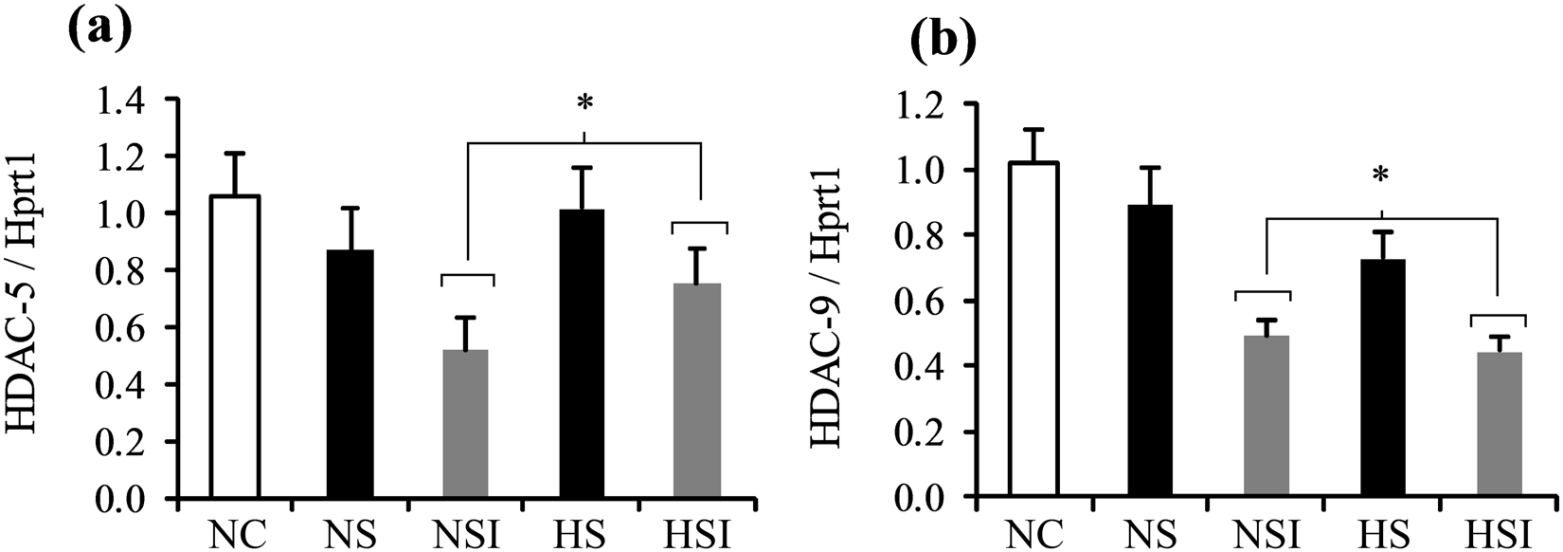
Effects of pulmonary inflammation and hypoxia exposure on mRNA contents of HDAC-5 (**a**) and HDAC-9 (**b**) in soleus muscle subjected to an overload surgery. (n = 5–6; Mean ± SEM); Hprt1 was used as housekeeping gene. NC: Normoxia Control; NS: Normoxia + Surgery; NSI: Normoxia + Surgery + Inflammation; HS: Hypoxia + Surgery; HSI: Hypoxia + Surgery + Inflammation. *Global effect of inflammation (NSI + HSI) *vs*. (NS + HS), p < 0.05.

We then questioned whether epigenetic modifications could be involved in the muscle hypertrophic capacity of animals subjected to the two main stimuli present in COPD (i.e. PI and CH). We first considered global histone acetylation as a readout for cellular epigenetic states. The histone H3 acetylation level was significantly higher in groups with inflammation (NSI + HSI) compared to the groups without (NS + HS, +33%, p < 0.05; Supplementary Figure 2 and Fig. 5a,b). This acetylation level also negatively correlated with the soleus muscle weight after surgery, as shown in Fig. 5c (r = 0.656, p < 0.001). Such an increase in histone H3 acetylation can be related to the decreased transcription levels of HDAC-9 and HDAC-5 mRNA (Fig. 6a,b). Indeed, HDAC-5 mRNA content decreased in the groups with PI (NSI + HSI) compared to the groups without administration (−32% *vs*. NS + HS, p < 0.05, Fig. 6a). The same pattern was observed for the mRNA levels of HDAC-9 which are globally lower after surgery when compared to the groups without PI (−42% *vs*. NS + HS, p < 0.05, Fig. 6b).

Altogether these experiments suggest that PI alters transcription of some HDACs that could explain the acetylation increase observed on the histone 3.

### Effect of Bromodomain and Extra-Terminal (BET) bromodomains inhibition

**Figure 7:**
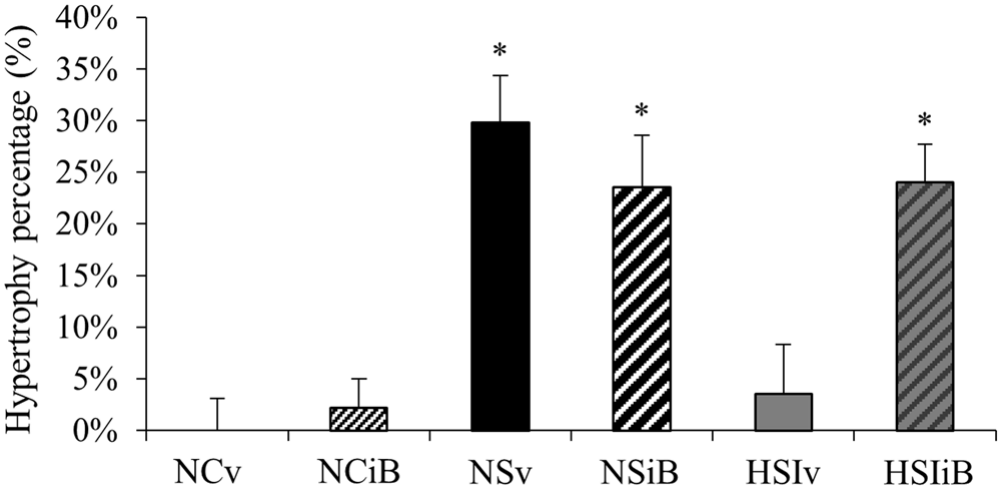
Effects of pulmonary inflammation + hypoxia exposure and I-BET151 administration on the hypertrophic capacities of soleus muscle subjected to an overload surgery. Hypertrophic capacity was determined by the ratio between soleus weight normalized with tibia length and the mean of the control group. (n = 7–8; Mean ± SEM); NCv: Normoxia control + vehicle; NCiB: Normoxia control + IBET; NSv: Normoxia + Surgery + vehicle; NSiB: Normoxia + Surgery + IBET; HSIv: Hypoxia + Surgery + Inflammation + vehicle; HSIiB: Hypoxia + Surgery + Inflammation + IBET. *Different *vs*. NCv, NCiB and HSIv, p < 0.05.

Hypertrophic capacities of soleus muscle after overload surgery were also explored after administration of an inhibitor of Bromodomain and Extra-Terminal bromodomains (I-BET151), a drug that specifically targets epigenetic signaling. This drug inhibits interactions between acetylated histones and Brd-containing effector proteins. The percentage of soleus muscle hypertrophy in groups treated only with vehicle showed the same pattern as those presented in Fig. 2 (Fig. 7), with an increase in normoxia (+30%, p < 0.05) and no significant change in hypoxia associated with inflammation (+3.6%, non significant). Treatment with I-BET151 in normoxia did not alter the hypertrophic response after surgery as present in vehicle controls. On the contrary, I-BET151 administration in hypoxia induced a hypertrophic response after surgery (+24% *vs*. NC, p < 0.05) which was absent in vehicle controls (+6%, non significant) and similar to the hypertrophy determined in normoxia after surgery.

**Figure 8:**
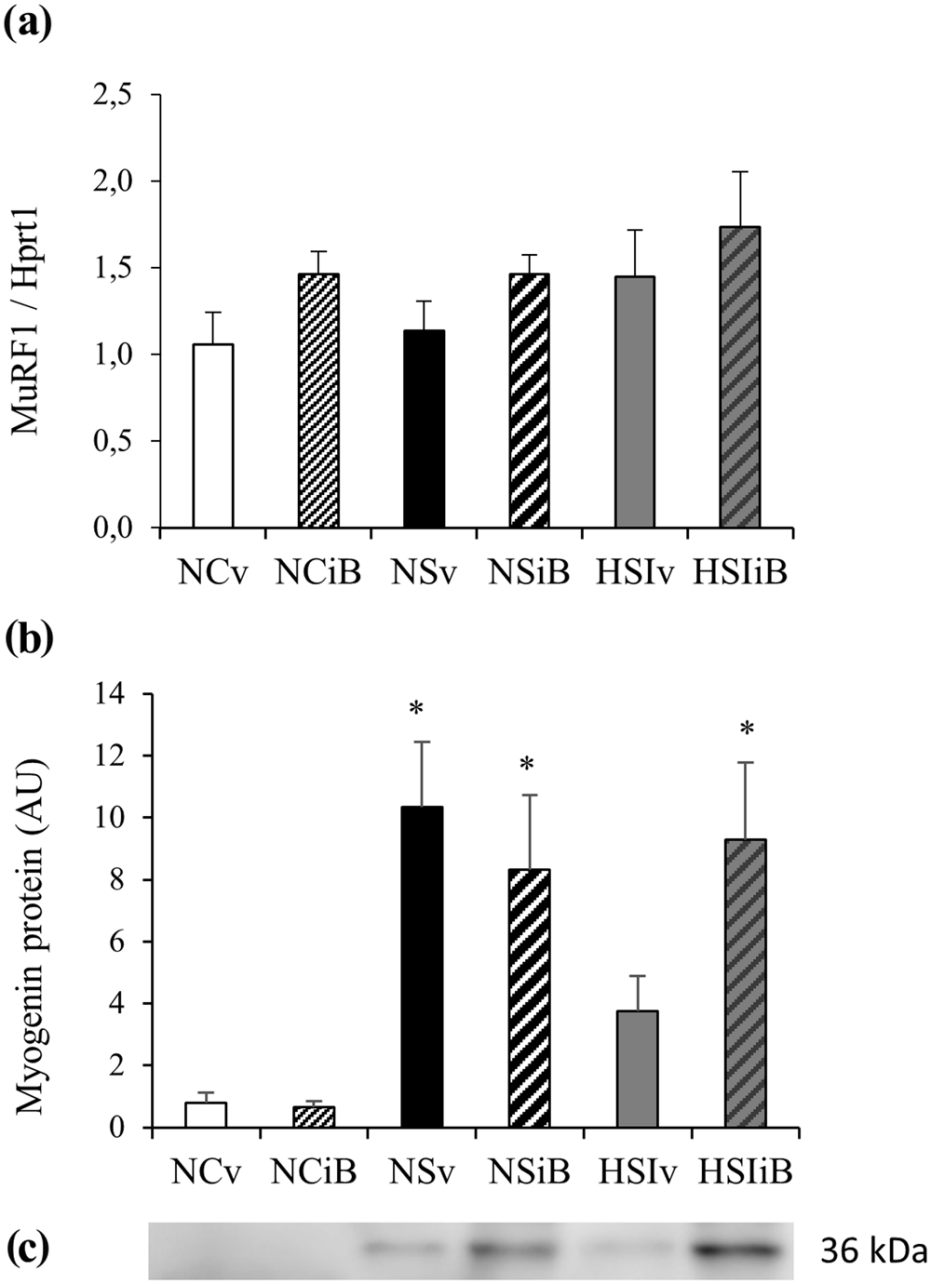
Effects of pulmonary inflammation + hypoxia exposure and I-BET151 administration on the MuRF-1 mRNA contents determined by RT-PCR (**a**) and the myogenin contents (**b**) determined by Western blot (**c**), in soleus muscle subjected to an overload surgery. (n = 5–8; Mean ± SEM); Hprt1 was used as housekeeping gene. NCv: Normoxia control + vehicle; NCiB: Normoxia control + IBET; NSv: Normoxia + Surgery + vehicle; NSiB: Normoxia + Surgery + IBET; HSIv: Hypoxia + Surgery + Inflammation + vehicle; HSIiB: Hypoxia + Surgery + Inflammation + IBET. *Different *vs*. NCv, NCiB, p < 0.05. Blot image has been cropped to the area of interest. The full-length blot is presented in Supplementary Figure 3.

Proteosynthesis and proteolysis regulatory factors such as the the contents of MuRF-1 mRNA and of myogenin protein were also determined (Fig. 8). I-BET151 did not significantly alter the transcription level of MuRF-1 mRNA in any of the experimental conditions (Fig. 8a). Myogenin content was not altered by I-BET151 in groups without overload surgery ( + 9%, NCv *vs*. NCiB, non significant). However, in groups subjected to overload surgery in normoxia, myogenin content was significantly increased with (NSiB) or whithout I-BET151 (NSv) when compared to controls (NCv) (respectively +28% and +33%, p < 0.05; Fig. 8b,c). In hypoxia associated with inflammation, myogenin content did not increase after the overload surgery (+15% HSIv *vs*. NCv, non significant), but was higher if I-BET151 was administered to the animals (+38% for HSIiB *vs*. NCv, p < 0.05, Fig. 8b,c).

## Discussion

This study shows that the hypertrophic capacity of the soleus muscle in response to an overload surgery is selectively sensitive to pulmonary inflammation with or without chronic hypoxia. These effects occur at the whole muscle level (weight), as well as at protein (proteosynthesis/proteolysis balance) and transcriptional levels (MuRF-1 mRNA). Moreover, we report for the first time that the inhibition of hypertrophy in the presence of inflammation is totally reversed by the administration of the inhibitor I-BET151, suggesting an involvement of histone acetylation-dependent signaling pathways.

To mimic muscle response to strength training, a modality more and more incorporated in exercise rehabilitation programs, we chose the model of muscle hypertrophy induced by surgical ablation of agonist muscles. The hypertrophy classically observed in response to the surgical overload^37^ that we observed in groups without PI could result from an activation of the proteosynthesis pathways because of the reported increase in myogenin content, considered to be associated with increase in proteosynthesis^38^. The situation seems quite different in groups where PI was performed. The significant increase of MuRF-1 mRNA transcription, as indicator for catabolic pathways^39^ and the similar level in myogenin in animals that underwent PI, despite the surgery, suggest that the proteosynthesis/proteolysis balance has not been modified and could not thus induce muscle mass increase. This specific muscle mass control in response to PI is yet not fully understood. In COPD, it has been suggested that PI could have repercussions in the blood compartment according to the over-spill concept^40^. We can speculate that a messenger links the PI to the muscle growth inhibition through a pathway different from those involving the classical proteins of inflammation. Further experiments are needed to clarify this. The weight loss observed in animals under hypoxia, inflammation or I-BET151 administration (Supplementary Table 2), seems to have no impact on the soleus muscle hypertrophy response as a similar weight loss in HS and HSI animals was associated with either soleus hypertrophy (HS group) or no response (HSI). Moreover, weight loss is a common feature of hypoxia exposure even with pair-feeding^10^. Similarly, despite a weight loss in I-BET151-treated animals, soleus response to overload surgery was not consistent between groups, so differences cannot be attributable to that parameter but merely to the treatment itself.

Chronic hypoxia as compared to normoxia did neither affect the hypertrophy response of soleus nor direct readouts of the proteosynthesis/lysis balance such as myogenin, MuRF-1 or S6K1 phosphorylation. However, it induced some changes in upstream signaling pathways suggesting that in normoxia and hypoxia, different mechanisms operate to trigger hypertrophy. In comparison to the normoxic groups, hypoxia inhibited Akt signaling in both absence and presence of inflammation, while it strongly activated MAP kinase signaling via Erk 1 and Erk 2, but only in absence of inflammation. Both Akt and MAP kinase pathways merge to activate mTORC1 and downstream S6K1 to finally stimulate proteosynthesis^41^. Thus, our data suggest that the hypertrophic response under normoxia is primarily controlled by activation of Akt and possibly other pathways, while this response under chronic hypoxia would be much more controlled by activation of Erk 1-2, thus compensating for Akt inhibition. Only the inhibition of Akt, but not Erk 1-2 activation, was maintained under inflammation, suggesting an inflammatory control of Erk signaling.

Pulmonary inflammation prevented the hypertrophy response of soleus muscle instead of reducing normal muscle mass. Indeed, we measured muscle mass evolution without overload surgery but in presence of either inflammation (NCI) or hypoxia (HC) alone or together (HCI). Results are presented as supplemental data (Supplementary Figure 2) and show that there is no effect of these conditions on non-challenged muscle. This lack of hypertrophic response could be linked to higher proteolysis, as judged by increased MuRF-1 transcription, and unchanged proteosynthesis, as judged by myogenin levels, both suggesting a modified proteosynthesis/proteolysis balance in favor of catabolism. At the molecular level, a lack of anabolic stimulation is confirmed by the unchanged S6K1 activation state. This non-response of S6K1 is only partially explained by changes in upstream Akt and Erk 1-2 signaling, since Akt phosphorylation is unchanged with and without inflammation, and Erk 1-2 only plays a role under additional hypoxia, as discussed above.

The control of muscle mass by inflammatory signaling is not yet fully understood, but likely involves a master regulator of muscle mass balance. Epigenetic alterations such as changes in histone acetylation have been repeatedly found in muscles of COPD patients^17,42^, and HDACs have been involved in proteosynthesis mechanisms^43^. Our study reveals an increase of histone H3 acetylation in the pooled inflammation groups, reaching levels found in the non-surgery control. This increase strongly correlates with reduced HDAC-5 and HDAC-9 mRNAs in the same groups, suggesting decreased histone deacetylation as a contributing mechanism. In support to this, decreased HDAC activity has been already associated with muscle atrophy in response to denervation^25^. Thus, it is likely to be also a main factor regulating the hypertrophic response of muscles to overload, although true causality still has to be shown.

To further examine the role of histone acetylation, we applied a pharmacological approach, using I-BET151^30,44^. This drug inhibits interaction of proteins with acetylated histones, thus interrupting epigenetic signaling. BET inhibitors have been first developed in cancer studies^45,46^. However, new usage can be considered in situations where inflammation is involved^24^. These BET inhibitors are being progressed through about 25 clinical trials so far (see ClinicalTrials.gov) and could be an interesting new therapeutic approach in the context of muscle mass loss in respiratory diseases. As COPD is better characterized by the combination of hypoxia and inflammation, we chose to study the I-BET effect in the presence of these two stimuli associated together in a second subset of experiments to be as relevant as possible with the context of the disease. I-BET151 was able to completely restore the hypertrophic response under conditions of hypoxia and inflammation to levels similar to those seen under normoxic, non-inflammatory control conditions. Measurements of myogenin protein content and MuRF-1 mRNA content suggest that the I-BET could act on the efficiency of the proteosynthesis without affecting the proteolysis pathway in animals exposed to hypoxia and pulmonary inflammation. This strongly supports a key role of histone acetylation in the response to chronic exposure to at least one of the two stimuli, hypoxia and/or inflammation. A role of acetylation in inflammatory pathways was already reported earlier by Nicodeme *et al*.^24^. Interestingly, I-BET151 does not have effect on muscle hypertrophy, myogenin protein or MuRF1 mRNA content in sham animals or after overload surgery in normoxia. This observation suggests that I-BET151 does not disturb all the transcriptional pathways of the muscular cell, but seems to control rather those involved in the muscle hypertrophy of animals subjected to pulmonary inflammation.

## Conclusion

This study shows that adaptive hypertrophy in response to a muscle overload surgery is differentially affected by inflammation and oxygen availability. Chronic hypoxia seems to maintain hypertrophy, although via signaling pathways different from normoxia. Pulmonary inflammation totally suppresses the hypertrophy response, linked to reduced HDACs mRNAs and increased histone H3 acetylation. Inhibitor of epigenetic signaling, using I-BET151 is able to completely restore the hypertrophic capacity under combined hypoxia and inflammation, providing clear evidence for epigenetic regulation of hypertrophy in oxidative muscles. These data provide a rationale for the failure of certain COPD patients to respond to a retraining program and open new perspectives in the medical care of these patients by using I-BET151.

## Electronic supplementary material

Supplemental data
