# Supplementary material for: Muscle hypertrophy in hypoxia with inflammation is controlled by bromodomain and extra-terminal domain proteins
Source: Sci Rep. 2017 Sep 21;7:12133. doi: 10.1038/s41598-017-12112-0 (PMC5608715; doi:10.1038/s41598-017-12112-0)
Supplement: Supplementary file 1 — Supplemental data [file 41598_2017_12112_MOESM1_ESM.doc]

**Supplemental information**

**Muscle hypertrophy in hypoxia with inflammation is controlled by Bromodomain and Extra-terminal domain proteins.**

Clovis Chabert^1^, Saadi Khochbin^2^, Sophie Rousseaux^2^, Rebecca Furze^3^, Nicholas Smithers^3^, Rab Prinjha^3^, Uwe Schlattner^1^, Christophe Pison^4^ and Hervé Dubouchaud^1^

^1^Univ. Grenoble Alpes, Inserm, Laboratoire de Bioénergétique Fondamentale et Appliquée (LBFA) Grenoble 38000 France

^2^Univ. Grenoble Alpes, Inserm, CNRS, Institute for Advanced Biosciences (IAB) Grenoble 38000 France

^3^Epigenetics DPU, Immuno-Inflammation Therapy Area, Medicines Research Centre GlaxoSmithKline R&D StevenageSG1 2NY England UK

^4^Univ. Grenoble Alpes, Inserm, CHU des Alpes, Laboratoire de Bioénergétique Fondamentale et Appliquée (LBFA) Grenoble 38000 France

Corresponding Author:

Hervé Dubouchaud, Laboratoire de Bioénergétique Fondamentale et Appliquée, INSERM U1055, Université Grenoble Alpes, CS 40700, 38058 Grenoble cedex 09, France.

Tel. +33 476 635 821; Fax +33 476 514 218

email: [herve.dubouchaud@univ-grenoble-alpes.fr](mailto:herve.dubouchaud@univ-grenoble-alpes.fr)

**Supplemental Materials and methods**

**Muscle overload model**

Animals were anesthetized by intra-peritoneal injection of xylazine/ketamine (8 mg/kg and 100 mg/kg, respectively). The bilateral ablation of the agonist muscles of the soleus was performed as described by Morioka *et al.* [^1^](#_ENREF_1). Sham operations were also performed under the same conditions. Animals were monitored until their total awakening and those from CH groups were replaced in the hypoxia chamber 24 h after surgery. Paracetamol diluted in the drinking bottle (300 mg/L) was delivered to animals during the seven days post-surgery.

**Protein electrophoresis and immunoblotting**

About 30 mg of frozen soleus were homogenized in 1 mL of 20 mM HEPES, 2 mM EGTA, 50 mM β-glycerophosphate, 3 mM benzamidine, 10% glycerol, 1% Triton X-100, 1mM DTT, 1mM Na_3_V0_4_, pH 7.4, supplemented with proteases inhibitor cocktail. Homogenization were performed using a ball mill (MM301 ; Retsch™, Düsseldorf, Germany). Protein were separated using a mini-PROTEAN II© system (Biorad™, Marne-La-Coquette, France), in SDS-PAGE gels and subsequently transferred on nitrocellulose membrane using standard conditions. Stacking gel was composed of 125 mM Tris-HCl, pH 6.8, 0.1 % (w/v) SDS, 6 % acrylamide/bis-acrylamide (37.5:1), 0.4 % (w/v) Ammonium persulfate, 0.05 % (v/v) TEMED. Separating gels were composed of 375 mM Tris-HCl, pH 8.8), 0.1 % (w/v) SDS, 7.5 % acrylamide/bis-acrylamide (37.5:1) except for the histone analysis where acrylamide/bis-acrylamide was at 20 %, 0.4 % (w/v) Ammonium persulfate, 0.05 % (v/v) TEMED. Primary antibodies against either S6K1, phospho-S6K1^thr389^, Erk1-2, phospho-Erk1-2^thr202/tyr204^, Akt, phospho-Akt^ser473^ (respectively: #2708 (RRID:AB_390722), #9205S (RRID:AB_330944), #9102 (RRID:AB_330744), #4377 (RRID:AB_331775), #4691 (RRID:AB_915783), #9271 (RRID:AB_329825), Cell Signaling Technology™, Danvers, Massachusetts, USA) diluted at 1:1000, or against myogenin (sc-12732 (RRID:AB_627980), Santa Cruz Biotechnology™, Dallas, Texas, USA) diluted at 1:250, were incubated overnight at 4°C after a 1h-membrane saturation in TBS, pH 7.4, 0.01 % (v/v) tween 20, 5 % (w/v) non fat milk. Appropriate secondary antibodies coupled with HRP diluted at 1:3000 (anti-rabbit NA934 (RRID:AB_772206), GE Healthcare™, Little Chalfont, England or anti-mouse 554002 (RRID:AB_395198), BD Pharmingen™, Franklin Lakes, New Jersey, USA) were incubated at room temperature for 1h. Reactions were revealed using enhanced chemiluminescence and signals were acquired on a LAS4000 imaging system (GE Healthcare, Little Chalfont, UK). Band intensities were quantified using Image J software. The mean value of the signals generated by all the samples from the NC group on each gel was used as an arbitrary unit to express the results from the other groups.

**RNA extraction and RT-qPCR**

Total RNA was purified from 20-25 mg of muscle homogenized in 1 mL of TRIzol® (ThermoFisher Scientific™, Waltham, Massachusetts, USA) using a ball mill. Total RNA purity and concentration were optically assessed using a nanophotometer (Classic, Implen™, München, Germany) at 230, 260 and 280 nm. Reverse transcription of 1 µg of total RNA was performed using 100 U of M-MLV reverse transcriptase in a final volume of 20 µL according to manufacturer's instructions (Superscript III, Invitrogen) using oligo-DTs as primers. Relative contents of target mRNA were determined by real-time measurements with a Fast Start DNA Master SYBR Green kit (Roche Applied Science™, Bâle, Sweiss) using a LightCycler (Roche Applied Science). Primers used were designed on ProbeFinder 2.50 (Roche Applied Science) and reported in Suppl. Table 1.

**Suppl. Table 1:** Primers sequences used during this study.

| **Genes** | **Primers sequences** | | **Amplicon length** | **Annealing temp (°C)** |
| --- | --- | --- | --- | --- |
| Hprt1 | 5'-ggtccattcctatgactgtagatttt-3' | 5'-caatcaagacgttctttccagtt-3' | 126 | 53 |
| MuRF1 | 5'-agcattgtagaagcttccaagg-3' | 5'-gtaaactcctcctcctcatctgtc-3' | 146 | 56 |
| HDAC-5 | 5'-cctggtttctgctggatttg-3' | 5'-tcgtcaaatgaccaaagcat-3' | 92 | 55 |
| HDAC-9 | 5'-tgcccttctaggaaatgagc-3' | 5'-agcggctgcattcgtatt-3' | 76 | 53 |

Hprt1: hypoxanthine phosphoribosyltransferase 1; MuRF1: Muscle ring Factor 1; HDAC-5 and 9: Histones Deacetylases-5 and 9.

**Supplemental data**

**Suppl. Table 2:** Morphological data of animals from the model development study (A) and the I-BET testing study (B).

| **A** |  |  | | | | | | | | | | | | | | |  |  |  |
| --- | --- | --- | --- | --- | --- | --- | --- | --- | --- | --- | --- | --- | --- | --- | --- | --- | --- | --- | --- |
|  |  | **NC** | | | **NS** | | | **NSI** | | | **HS** | | | **HSI** | | |  |  |  |
|  |  | *(n = 8)* | | | *(n = 7-8)* | | | *(n = 7)* | | | *(n = 7)* | | | *(n = 7-8)* | | |  |  |  |
| **Initial body weight** | (g) | 413 | ± | 12.1 | 429 | ± | 8.0 | 437 | ± | 9.7 | 426 | ± | 3.9 | 450 | ± | 5.2 |  |  |  |
| **Final body weight** | (g) | 441 | ± | 15.8 | 429 | ± | 4.8 | 429 | ± | 14.6 | 388 | ± | 11.4 † | 400 | ± | 6.1 † |  |  |  |
| **Tibial Length** | (cm) | 4.41 | ± | 0.05 | 4.31 | ± | 0.02 | 4.36 | ± | 0.02 | 4.50 | ± | 0.06 | 4.30 | ± | 0.03 |  |  |  |
| **Soleus weight** | (mg) | 152.9 | ± | 5.3 | 182.6 | ± | 9.1* | 143.9 | ± | 5.9 | 192.5 | ± | 6.8* | 148.5 | ± | 5.3 |  |  |  |
|  |  |  |  |  |  |  |  |  |  |  |  |  |  |  |  |  |  |  |  |
|  |  |  |  |  |  |  |  |  |  |  |  |  |  |  |  |  |  |  |  |
| **B** |  |  | | | | | | | | | | | | | | | | | |
|  |  | **NCv** | | | **NCiB** | | | **NSv** | | | **NSiB** | | | **HSIv** | | | **HSIiB** | | |
|  |  | *(n = 8)* | | | *(n = 7)* | | | *(n = 8)* | | | *(n = 8)* | | | *(n = 7-8)* | | | *(n = 7-8)* | | |
| **Initial body weight** | (g) | 452 | ± | 6.6 | 442 | ± | 7.7 | 459 | ± | 7.2 | 447 | ± | 8.1 | 450.0 | ± | 2.4 | 448 | ± | 9.4 |
| **Final body weight** | (g) | 428 | ± | 8.4 | 434 | ± | 11.2 | 442 | ± | 8.7 | 385 | ± | 11.8 | 373.4 | ± | 4.5 † | 322 | ± | 7.6 † |
| **Tibial Length** | (cm) | 4.38 | ± | 0.02 | 4.41 | ± | 0.03 | 4.41 | ± | 0.04 | 4.38 | ± | 0.02 | 4.43 | ± | 0.04 | 4.34 | ± | 0.04 |
| **Soleus weight** | (mg) | 146.3 | ± | 4.6 | 155.3 | ± | 3.9 | 195.5 | ± | 7.0^ll^ | 183.4 | ± | 5.6^ll^ | 151.7 | ± | 0.04 | 4.34 | ± | 7.1^ll^ |
|  |  |  |  |  |  |  |  |  |  |  |  |  |  |  |  |  |  |  |  |

(n = 7-8; Mean ± SEM). NC: Normoxia Control; NS: Normoxia + Surgery; NSI: Normoxia + Surgery + Inflammation; HS: Hypoxia + Surgery; HSI: Hypoxia + Surgery + Inflammation; NCv: Normoxia control + vehicle; NCiB: Normoxia control + IBET; NSv: Normoxia + Surgery + vehicle; NSiB: Normoxia + Surgery + IBET; HSIv: Hypoxia + Surgery + Inflammation + vehicle; HSIiB: Hypoxia + Surgery + Inflammation + IBET. †: global effect of hypoxia, p<0.05; *: global effect of inflammation, p<0.05; ll: diff. of NCv, p<0.05.


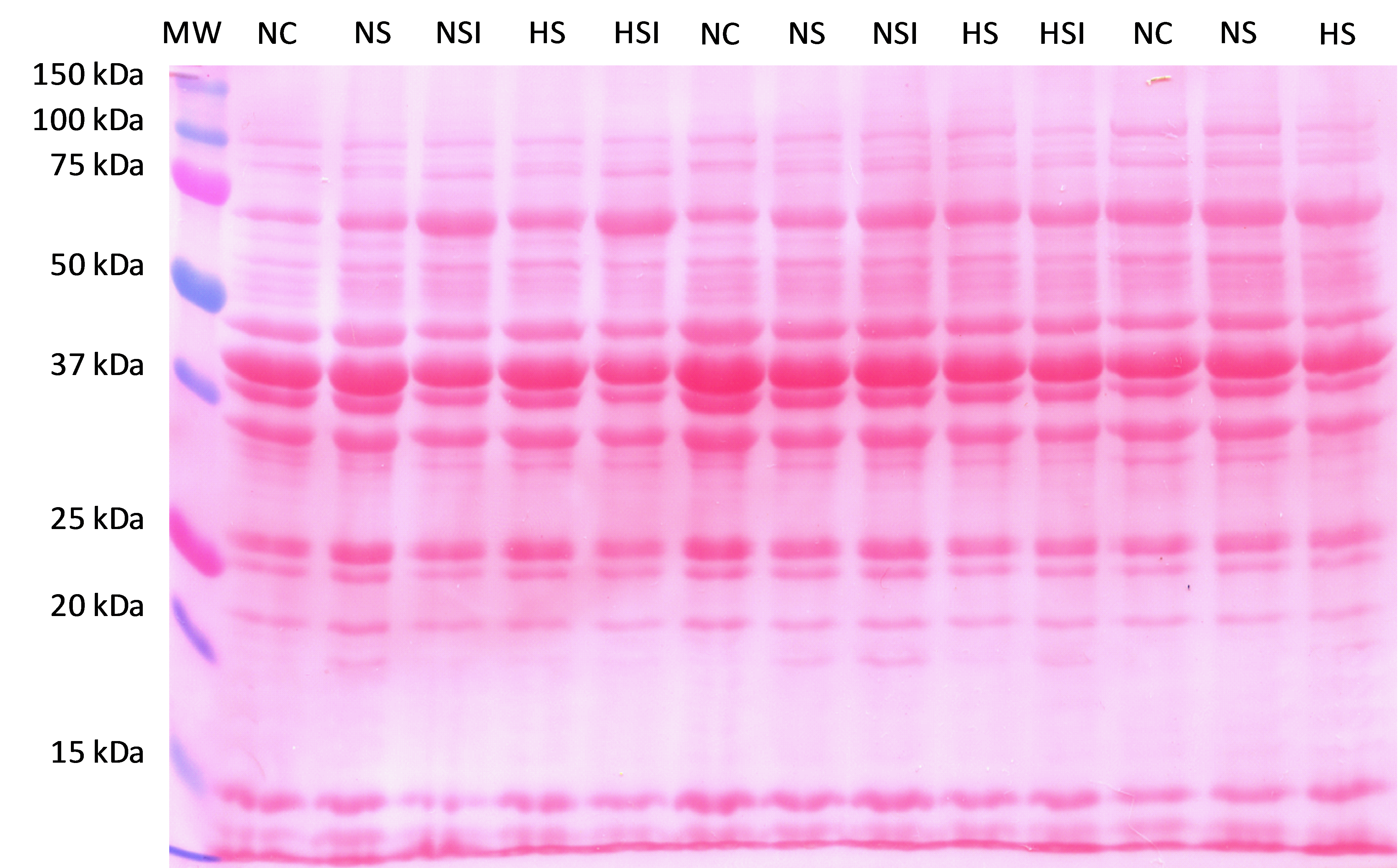


**Supplemental Figure. 1:** Example of a digitized membrane after a Ponceau S staining.

NC: Normoxia control; NS: Normoxia + Surgery; NSI: Normoxia + Surgery + LPS administration; HS: Hypoxia + Surgery; HSI: Hypoxia + Surgery + LPS administration.


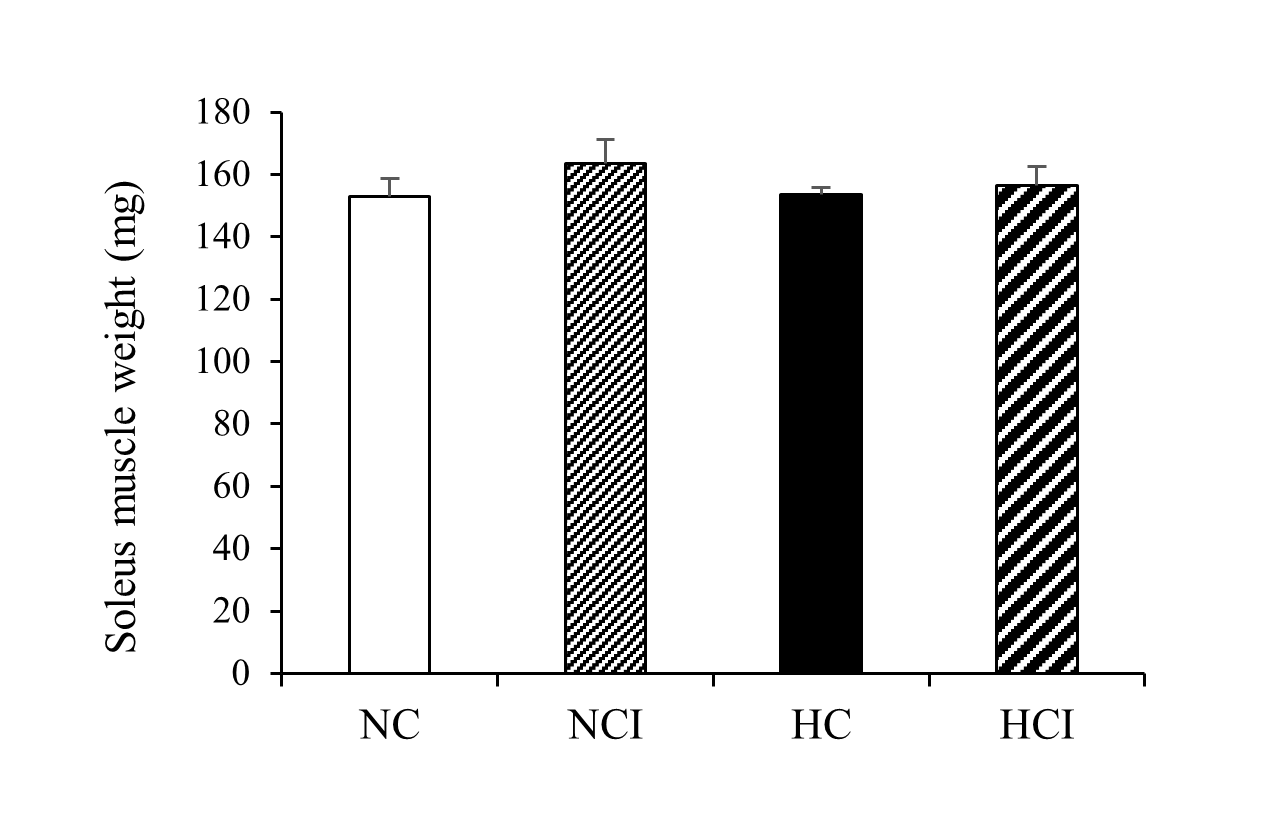


**Supplemental Figure 2:** Effects of pulmonary inflammation and hypoxia exposure on the soleus muscle mass. NC: Normoxia Control; NCI: Normoxia Control + Inflammation; HC: Hypoxia Control; HCI: Hypoxia Control + Inflammation.

**References**

1 Morioka, S. *et al.* Functional overloading facilitates the regeneration of injured soleus muscles in mice. *The journal of physiological sciences : JPS* **58**, 397-404, doi:10.2170/physiolsci.RP004008 (2008).


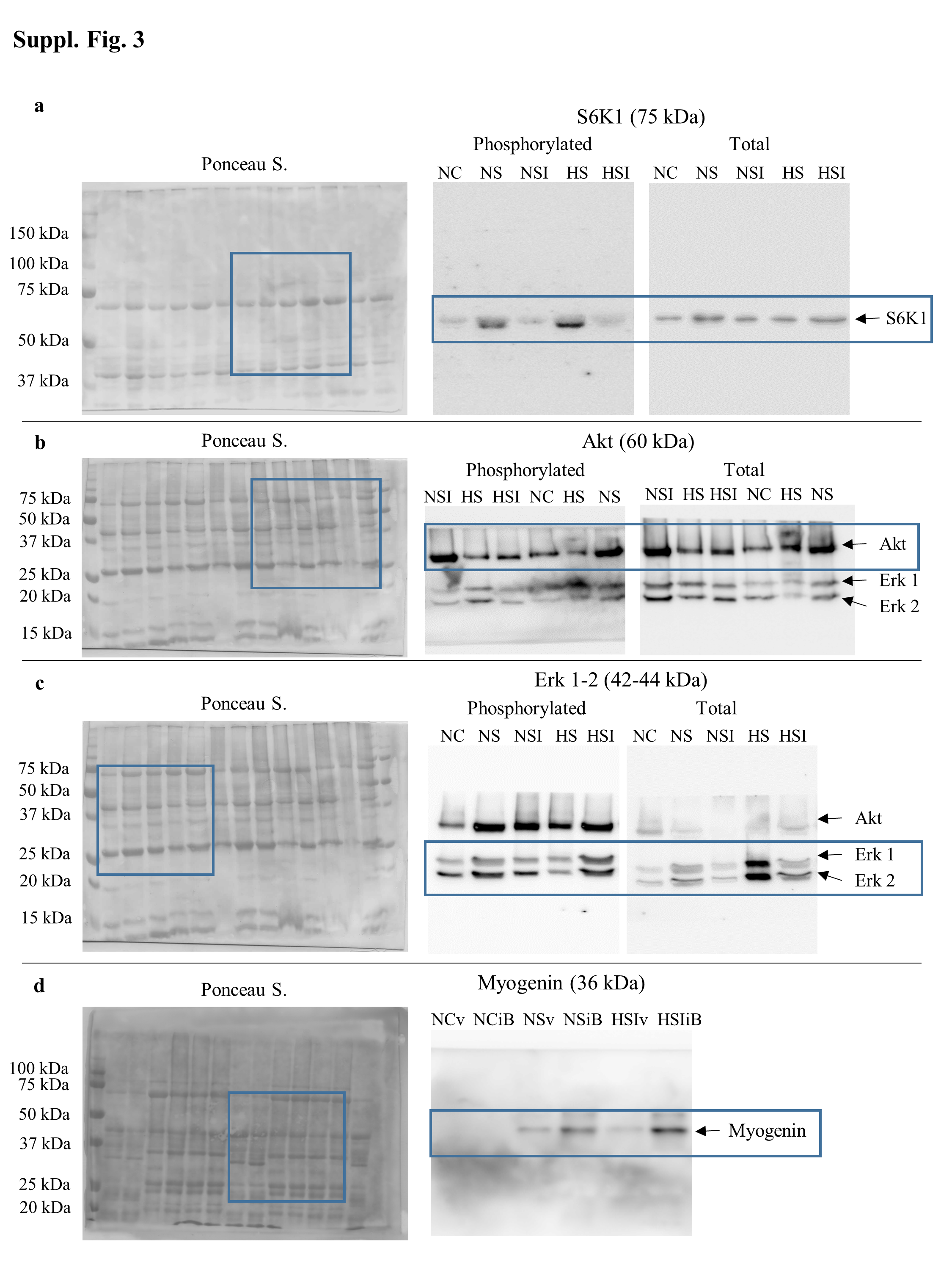


**Supplemental Figure 3:** Original full-length blots and Ponceau S-stained membranes. Frames delineate the portion used to prepare the figures.
